# The impact of civil commitment laws for substance use disorder on opioid overdose deaths

**DOI:** 10.3389/fpsyt.2024.1283169

**Published:** 2024-02-02

**Authors:** Phillip Cochran, Peter S. Chindavong, Jurian Edelenbos, Amy Chiou, Haylee F. Trulson, Rahul Garg, Robert W. Parker

**Affiliations:** Alabama College of Osteopathic Medicine, Dothan, AL, United States

**Keywords:** civil commitment, COVID-19 pandemic, opioid use disorder (OUD), substance usage disorders (SUDs), involuntary commitment, United States

## Abstract

**Objective:**

Our study analyzed the impact of civil commitment (CC) laws for substance use disorder (SUD) on opioid overdose death rates (OODR) in the U.S. from 2010–21.

**Methods:**

We used a retrospective study design using the CDC Wide-ranging Online Data for Epidemiologic Research (WONDER) dataset to analyze overdose death rates from any opioid during 2010–21 using ICD-10 codes. We used *t*-tests and two-way ANOVA to compare the OODR between the U.S. states with the law as compared to those without by using GraphPad Prism 10.0.

**Results:**

We found no significant difference in the annual mean age-adjusted OODR from 2010–21 between U.S. states with and without CC SUD laws. During the pre-COVID era (2010–19), the presence or absence of CC SUD law had no difference in age-adjusted OODR. However, in the post-COVID era (2020–21), there was a significant increase in OODR in states with a CC SUD law compared to states without the law (*p* = 0.032). We also found that OODR increased at a faster rate post-COVID among both the states with CC SUD laws (*p* < 0.001) and the states without the law (*p* = 0.019).

**Conclusion:**

We found higher age-adjusted OODR in states with a CC SUD law which could be due to the laws being enacted in response to the opioid crisis or physicians’ opposition to or unawareness of the law’s existence leading to underutilization. Recent enactment of CC SUD law(s), a lack of a central database for recording relapse rates, and disparities in opioid overdose rate reductions uncovers multiple variables potentially influencing OODR. Thus, further investigation is needed to analyze the factors influencing OODRs and long-term effects of the CC SUD laws.

## 1 Introduction

In 2019, the United Nations Office on Drugs and Crime (UNODC) reported that over 1.92 million people received treatment for drug use in the U.S. (1) More than 43.2% of that group received treatment for opioid use, which was the predominant drug class over hallucinogens, cocaine, cannabis, solvents and inhalants, and sedatives and tranquilizers (1). In 2020, 4.56% of the U.S. population (~15.1 million people) used opioids which included prescription opioids (1, 2). Recently, opioid dispensing rates have decreased in the U.S. overall; however, Alabama, Arkansas, Kentucky, Louisiana, Mississippi, and Tennessee remain above the national average (3). For example, Alabama showed, in 2018, a rate of 97.5 opioid prescriptions (Rx) per 100 people, higher than the national average of 51.4 Rx per 100 people. This rate decreased from 142 Rx per 100 people, reported in 2012. By 2020, Alabama was still highest among all states at 80.4 Rx per 100 people (3). Further, strict criminal laws for drug possession may lead to adverse events among opioid users if medical care is not available (4). As the U.S. opioid dispensing rates decrease, people are turning to cheaper, more accessible and potent illicit drugs, such as synthetic opioids (5). Overdose deaths involving synthetic opioid use, particularly fentanyl use, have risen in the past decade (6). In 2016, fentanyl and its analogs contributed to nearly half of opioid overdose deaths in the United States (6). In 2019, the National Forensic Laboratory Information System crime laboratory data reported a 12% increase in fentanyl identification and a 13% decrease in heroin reports (5).

In 2017, there were 70,237 drug-related overdose deaths, with opioids being the primary drug, followed by cocaine and amphetamine-type stimulants, per the UNODC (1). By June 2021, drug overdose deaths increased to 100,569, which was an increase of 21.3% from the previous year’s 82,916 deaths. In February 2023, 105,258 drug overdose deaths were reported (7).

Civil commitment (CC) for substance use disorder (SUD) is a form of involuntary commitment (IC) that provides a legal process for a judicial court to place an individual with an SUD diagnosis in medically supervised treatment. If specific criteria, such as being “gravely disabled” or posing a threat to themselves or others are met due to cognitive deficiencies related to substance use then an individual could petition for CC for SUD (8–10). Interested parties, such as family, community members, healthcare professionals, government officials, etc., must file a petition with the court to initiate the process of CC for the specific individual (8, 9). Once filed, the individual named in the petition receives a copy of the petition and a notice to appear for a hearing (11). At the hearing, the presiding judge reviews the petition along with any presented evidence and determines whether the assertions provided in the petition are substantiated and whether the state’s statutory criteria for CC for SUD are met (8, 11). Not all states have CC laws for SUD (9). In states without a CC SUD law, a patient with SUD cannot be mandated by law to receive treatment (e.g., in-stay rehab, outpatient rehab, etc.) for SUD. As of 2021, 34 states and the District of Columbia (D.C.) have enacted CC laws for SUD (9). Each state has varying statutory requirements for CC for SUD. Some states may not explicitly require a hearing for CC for SUD, while it is common for IC for other mental illnesses. Nonetheless, all CC SUD laws specify certain criteria that each case must meet (12). Once the court determines that the statutory requirements are met, the individual is taken into custody and placed in the appropriate SUD treatment (9, 12). A study found amphetamine use, inhalant use, and a history of polysubstance use was significantly higher in mentally ill substance users when compared to a group of similar size of substance users without DSM-IV criteria mental illnesses (13). The study also found the mentally ill patients to have statistically higher addiction severity index scores in medical status (13). Whereas, composite scores for alcohol use, employment, and legal status did not significantly differ (14).

Insufficient data exists on the effectiveness of the CC law for SUD. In a 2013 Florida study on CC for SUD, 69% completed the CC program and 70% completed voluntary treatment facility admissions. This study demonstrates CC’s usefulness for SUD in addition to various complexities and how patient motivation can affect treatment outcomes. For instance, a patient pursuing treatment may have external motivators (e.g., fear of losing marriage or source of income, family pressure to enter treatment, and repercussions of criminal offenses) influencing one’s motivation to receive and/or complete treatment (15). States differ in CC laws for SUD, including but not limited to diagnostic criteria, treatment type (residential or outpatient), and mandated treatment duration, ranging from 2 weeks to 1 year. Nebraska, Iowa, Michigan, and D.C. have no defined maximum initial commitment duration; however, the treatment duration would still need to be specified upon commitment. Initial commitment duration ranges from 14 days to unspecified with 26 states and D.C. having a recommitment process with the CC SUD laws (9). The protocols also vary based on the type of mental illness, with most states that have a CC SUD law explicitly stating that the use of CC for SUD is authorized. Whereas some states use a broader term such as “mentally ill person” and then list substance use/intoxication etc. to authorize the use of CC. This minor difference is significant because a patient could have an SUD diagnosis but no mental illness or a lack of evidence that their ability to care for themselves is impaired. In most states, CC for SUD excludes invasive treatments such as medication injections. However, 12 U.S. states permit non-consensual medication under CC SUD laws. States differ in what is permitted under CC SUD law, which include: surgery (4 states), electric shock (1), restraint (13), seclusion (10) while 15 states and D.C. do not specify what is permitted. Variability in state CC laws for SUD makes demonstrating effectiveness challenging. A 2015 study found that Florida and Massachusetts had the highest usage of CC laws for SUD among all states, with Florida having >9,000 annual uses and Massachusetts >4,500 uses in 2011 (16). Wisconsin had the next highest, with 260 uses in 2011. CC SUD laws have the potential to curtail rapidly increasing opioid overdose deaths in the U.S. Given the lack of evidence on the effectiveness of these laws, our study investigated the impact of CC SUD laws on overall death rates due to any opioid overdose. In this study, we analyzed opioid overdose death rates (OODR) from 2010–21 in all 50 states and D.C. comparing the states that have CC SUD laws and those that do not. We hypothesized that states with a CC SUD law would have significantly lower OODR than states without a CC SUD law from 2010–21.

## 2 Methods

### 2.1 Study data

Centers for Disease Control and Prevention (CDC) Wide-ranging Online Data for Epidemiologic Research (WONDER) dataset was used to analyze OODR from 2010-21 for all 50 states and D.C. using Multiple Cause of Death (MCD) - International Classification of Diseases, Tenth Revision (ICD-10) codes: Drug poisonings (overdose) – Unintentional (X40-X44), Suicide (X60-64), Homicide (X85), and Undetermined (Y10-Y14) (17).

### 2.2 CDC WONDER data query parameters

The following parameters were used to retrieve data from CDC WONDER: MCD-ICD-10 - Drug/Alcohol induced causes: Drug poisonings (overdose) Unintentional (X40-X44); Drug poisonings (overdose) Suicide (X60–X64); Drug poisonings (overdose) Homicide (X85); Drug poisonings (overdose) Undetermined (Y10–Y14).

Group by: state.
Calculate rates per: 100,000.
Demographics: all ages, all genders, all races, all origins.
Autopsy: all values.
Place of death: all.
Boxes checked for: age-adjusted rate, 95% confidence interval, standard error, percent of total deaths.

Data from CDC WONDER (2010–21) was exported as text files, then converted to Excel. Data was retrieved June 2023; therefore, provisional data for 2022 and partial/provisional data for 2023 were excluded. The *t*-tests and two-way ANOVA were performed in GraphPad Prism 10.0.

### 2.3 Data measures

Age-adjusted death rates were used rather than crude death rates to control for the effect of age on mortality. States were categorized based on the presence or absence of a state statute for CC for SUD. Arizona, Pennsylvania, and Wyoming were categorized as not having a CC SUD law because their laws only allow IC if a mental health disorder is diagnosed, not solely for SUD (9). Opioid-overdose deaths were classified into pre-COVID era (2010–19) and post-COVID era (2020–21). We also analyzed the OODR over the last decade (2012–21). Opioid-related deaths were aggregated by sex, age, and race/ethnicity per 100,000 persons.

### 2.4 Statistical analysis

A two-way analysis of variance (ANOVA) with Šídák testing (alpha <0.05) compared age-adjusted OODR across 4 groups: (1) U.S. states without a CC SUD law (2010–19), (2) U.S. states with a CC SUD law (2010–19), (3) U.S. states without a CC SUD law (2020–21), and (4) U.S. states with a CC SUD law (2020–21). The *p*-values were indicated as: ns (*p >* 0.05), *(0.01 ≤ *p <* 0.05), ****(*p <* 0.0001). The analysis assumed unequal variances, and generated slopes for each state during the years 2010–19, 2020–21, and the combined 10-year range from 2012–21. The OODR data for North Dakota was reported as unreliable for 2011 and, hence, was omitted from our study (17).

## 3 Results

**Figure 1:**
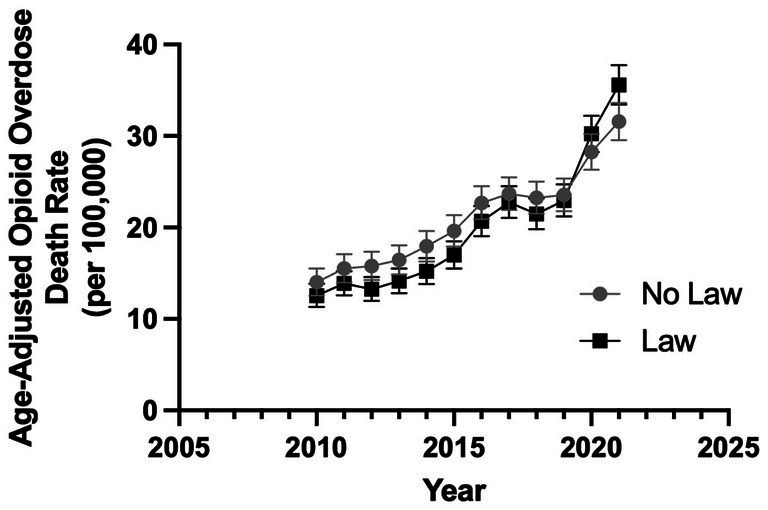
Annual age-adjusted OODR from 2010–21 comparing states with vs without civil commitment law. The error bars are 95% confidence interval (CI) of the mean (*t*-test comparing mean annual age-adjusted OODR for no law vs law, *p* = 0.35).

Table 1 presents the average slopes of age-adjusted OODR for different time periods. A *t*-test reveals no significant difference in the annual mean age-adjusted OODR between states without a CC SUD law and states with such a law from 2010–21 (*p* = 0.35). Prior to the COVID era (2010–19), the presence or absence of the CC SUD law did not significantly impact the slopes of age-adjusted OODR (*p* = 0.39, Table 1). However, after the COVID era (2020–21), there is a significant difference in the slopes of age-adjusted OODR when comparing states without the CC SUD law to those with it (*p* = 0.032, Table 1). Figure 1 illustrates the annual averages of age-adjusted OODR from 2010–21, comparing states with and without CC SUD law.

**Table 1:** Mean slopes (increase) of age-adjusted OODR comparing states with or without CC SUD law for years 2010–21 stratified into years 2010–19 and 2020–21.

Year range	No law (mean slope)	Law (mean slope)	*p*-value
2010–19 (Pre-COVID era)	1.21	1.32	0.39
2020–21 (Post-COVID era)	3.31	5.35	0.032
2012–21 (Last decade)	1.61	2.23	0.053

OODR, Opioid overdose death rates; CC, Civil commitment; SUD, Substance use disorder.

**Figure 2:**
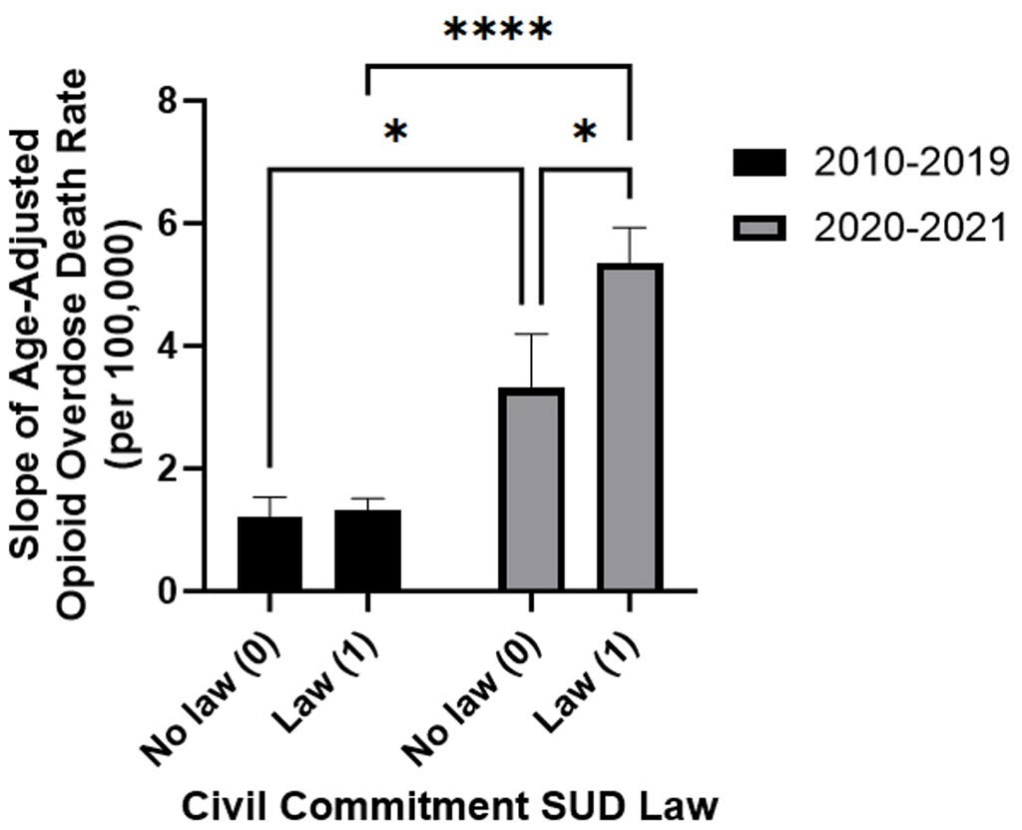
Slope of age-adjusted opioid overdose death rates (OODR) comparing 2010–19 to 2020–21 amongst states with or without civil commitment (CC) substance use disorder (SUD) law. Error bars display standard error of the mean (SEM) of age-adjusted OODR in states stratified by CC SUD law versus no CC SUD law states. Two-way ANOVA with Šídák testing confirmed year-range-dependent effects. *p*-value comparisons: ns (*p* > 0.05), *(0.01 ≤ *p* < 0.05), ****(*p* < 0.0001).

Although no statistical significance is found in OODR between states with and without CC SUD laws, from 2012–21, an increasing trend is evident amongst the slopes of states with and without a law (*p* = 0.053, Table 1; Figure 1). The OODR increased at a faster rate post-COVID (2020–21) as compared to pre-COVID among states without a CC SUD law (*p* = 0.019) and those with a CC SUD laws (*p* = 3.0×10^−8^) (Table 2). Table 2 displays the average slopes of OODR for states with and without CC SUD laws for years 2010–19 and 2020–21. Figure 2 presents a graph comparing the impact of CC SUD law on age-adjusted OODR between 2010–19 and 2020–21.

**Table 2:** Mean slopes (increase) of age-adjusted OODR for states with or without CC SUD law comparing years 2010–19 (Pre-COVID era) and 2020–21 (Post-COVID era).

	2010–19 (Pre-COVID era) Mean slope	2020–21 (Post-COVID era) Mean slope	*p*-value
No CC SUD law	1.21	3.31	0.019
CC SUD law	1.32	5.35	3.0 × 10^−8^

OODR, Opioid overdose death rates; CC, Civil commitment; SUD, Substance use disorder.

A two-way ANOVA with Šídák test was performed (Table 3). Supplementary Table S1 provides two-way ANOVA source of variation which yielded the following results: (1) years 2010-19 and 2020-21 do not have a consistent impact on OODR across all absence or presence of law values (*p* = 0.084), (2) the year ranges significantly affect the results (*p* < 0.0001), (3) absence or presence of law does not significantly impact the results (*p* = 0.054). Table 4 provides the slopes for each state stratified by 2010–19, 2020–21, and a 10-year range 2012–21.

**Table 3:** Two-way ANOVA of four groups: civil commitment law vs. no law and 2010–19 vs. 2020–21.

Šídák’s multiple comparisons test	Predicted (LS) mean difference	95% CI of difference	Below threshold?	Summary	Adjusted *p*-value
No CC SUD law states					
2010–19 vs. 2020–21	−2.1	−3.92 to −0.28	Yes	*	0.024
CC SUD law state					
2010–19 vs. 2020–21	−4.031	−5.26 to −2.80	Yes	****	<0.0001
Pre-COVID era 2010–19					
No law vs. Law	−0.1098	−1.66 to 1.44	No	ns	0.89
Post-COVID era 2010–19					
No law vs. Law	−2.041	−3.59 to −0.49	Yes	*	0.010

Test details	Predicted (LS) mean 1	Predicted (LS) mean 2	Predicted (LS) mean difference	SE of difference	*n*1	*n*2	*t*	df
No law								
2010–19 vs. 2020–21	1.21	3.31	−2.1	0.92	16	16	2.29	98
Law								
2010–19 vs. 2020–21	1.32	5.35	−4.03	0.62	35	35	6.51	98
2010–19								
No law vs. Law	1.21	1.32	−0.11	0.78	16	35	0.14	98
2020–21								
No law vs. Law	3.31	5.35	−2.04	0.78	16	35	2.61	98

Alpha set at 0.05. CI, Confidence interval; LS, Least Squares Means; SE, Standard error; *n*, sample size; *t*, t statistic; df, Degrees of freedom. *means (0.01 ≤ *p* < 0.05) and ****means (*p* < 0.0001).

Supplementary Table S2 provides annual age-adjusted OODR from 2010–21 comparing states with vs. without CC SUD law with confidence intervals. Supplementary Figures S1–S51 depict age-adjusted OODR for each U.S. state and D.C. from 2012–21.

**Table 4:** Slopes (increase in death rates) for each state stratified by 2010–19, 2020–21, and a ten-year range 2012–21.

State	CC SUD law status	Slope (2010–19)	Slope (2020–21)	Slope over ten years (2012–21)
Alabama	No law	0.700	7.710	1.453
Alaska	CC SUD law	0.476	13.650	1.344
Arizona	No law^a^	0.974	2.990	2.231
Arkansas	CC SUD law	0.322	3.180	0.916
California	CC SUD law	0.374	4.780	1.491
Colorado	CC SUD law	0.410	6.520	1.375
Connecticut	CC SUD law	2.973	3.190	3.325
Delaware	CC SUD law	3.710	6.730	4.582
District of Columbia	CC SUD law	3.995	5.580	5.830
Florida	CC SUD law	1.392	2.470	2.774
Georgia	CC SUD law	0.414	5.490	1.064
Hawaii	CC SUD law	0.475	−1.030	0.866
Idaho	No law	0.365	3.040	0.537
Illinois	No law	1.493	0.960	1.992
Indiana	CC SUD law	1.655	6.350	2.728
Iowa	CC SUD law	0.327	1.020	0.653
Kansas	CC SUD law	0.347	6.870	1.022
Kentucky	CC SUD law	1.318	6.440	3.029
Louisiana	CC SUD law	1.789	13.230	3.912
Maine	CC SUD law	2.751	7.370	3.618
Maryland	No law	3.610	−1.750	3.805
Massachusetts	CC SUD law	2.926	2.920	2.587
Michigan	CC SUD law	1.698	2.830	1.848
Minnesota	CC SUD law	0.659	5.510	1.414
Mississippi	CC SUD law	0.202	7.270	1.436
Missouri	CC SUD law	1.334	4.420	2.165
Montana	No law	−0.076	3.920	0.483
Nebraska	CC SUD law	0.126	0.060	0.457
Nevada	No law	−0.064	3.190	0.718
New Hampshire	No law	3.168	2.050	1.868
New Jersey	No law	2.787	0.330	2.648
New Mexico	No law	0.401	12.570	2.276
New York	No law	1.350	3.340	1.904
North Carolina	CC SUD law	1.452	8.260	2.577
North Dakota	CC SUD law	1.074	1.610	1.481
Ohio	CC SUD law	3.180	0.950	3.278
Oklahoma	CC SUD law	−0.182	5.030	0.027
Oregon	No law	0.020	8.120	1.139
Pennsylvania	No law^a^	2.994	0.810	2.937
Rhode Island	No law	1.864	3.560	2.175
South Carolina	CC SUD law	1.179	7.830	2.975
South Dakota	CC SUD law	0.341	2.340	0.585
Tennessee	CC SUD law	1.621	10.960	3.758
Texas	CC SUD law	0.125	2.650	0.663
Utah	No law	0.170	0.610	−0.320
Vermont	CC SUD law	1.867	9.450	2.953
Virginia	CC SUD law	1.332	3.900	2.160
Washington	CC SUD law	0.251	6.080	1.236
West Virginia	CC SUD law	3.128	9.520	6.043
Wisconsin	CC SUD law	1.210	3.890	1.855
Wyoming	No law^a^	−0.370	1.530	−0.166
Wisconsin	CC SUD law	1.210	3.890	1.855

a Unique clauses in CC laws led to states Arizona, Pennsylvania, and Wyoming being categorized as not having an SUD (https://pdaps.org/datasets/civil-commitment-for-substance-users-1562936854). These states only permit CC for a diagnosed mental health disorder and not solely on the condition of SUD.

## 4 Discussion

Our study showed a significant increase in the rates of OODR over the past decade. Surprisingly, we found that the rates of OODR increased at a faster rate among the states with CC SUD laws as compared to those without the CC SUD laws after the COVID-19 pandemic. It may be because the CC SUD laws were enacted in these states due to high overdose death rates as an emergency response. It needs to be seen if CC SUD laws are effective in the long term. This demonstrates a dramatic change in OODR in the U.S. that correlates with the pandemic. It could also be due to physicians’ opposition to or unawareness of the law’s existence leading to underutilization (18). This suggests that the states with a CC SUD law may have higher pre-existing age-adjusted OODR. Despite the data showing higher age-adjusted OODR in states with CC SUD laws from 2020–21, some states recently enacted such laws. The lack of central recording locations for data acquisition of states using CC for SUD and fluctuating timeframes of enactment dates demonstrates the need for further investigations to determine how CC SUD law has affected patients. As more data becomes available with time, it will be possible to assess changes in death rates and identify significant factors influencing the OODR in each state. An improvement to the study would be to analyze individual state age-adjusted OODR and compare them to the enactment dates of each state’s CC SUD law to identify if states are utilizing the laws and if the laws are effective in reducing age-adjusted OODR.

Historical abuse of IC has led to skepticism of CC for SUD in the medical community. Early 1900’s facilities committed patients without a diagnosed mental illness for extended periods of time which led to a loss of possessions and an infringement on patient autonomy (8, 19). Despite changes in mental healthcare practices and legal reform, a distressing past affecting one of the most vulnerable patient populations has kept concern for patient autonomy at the forefront of CC for SUD discussion. In addition, ambiguity in the literature arises from linking CC for SUD with outcomes in criminal cases. However, recent literature has uncovered mandated treatment for SUD can be effective, but more research is needed to better understand protective factors in treatment outcomes (20, 21). CC laws for SUD offer a treatment approach for patients and families at a crossroads who have exhausted other options in addition to serving as a preventive measure against criminality. Individuals in the community and in healthcare realize substance use influences an individual’s thought processes and behavior. Thus, CC for SUD has been reconstructed from policies that were originally used for severe mental illness, such as psychotic disorders, where a potential harm to self or others warranted intervention (12). However, patient autonomy and consideration for capacity are still valid concerns among the medical community in approaching SUD treatment. In sum, more research is needed to elucidate the impact and efficacy of CC SUD laws on relapse and drug overdose rates.

The surge in fatal opioid overdoses has led to community-based advocacy for mandatory treatment. In 2004, the mother of Mathew Casey Wethington was able to lobby for a CC SUD law in her home state of Kentucky after losing her son who died from a heroin overdose (22, 23). The opioid epidemic has led to shifting viewpoints of CC SUD laws and communities are advocating for change. Those changes to CC SUD laws include extensive criteria for commitment, follow-up court hearings, and physicians who are advocating for patients who are a danger to themselves or others (19, 24, 25). Yet there are varying opinions among physicians in related specialties. According to a study in 2007 that surveyed psychiatrists, there is less support for CC for drug and alcohol use (22%). Meanwhile, a 2021 survey reported 60.7% of Addiction Medicine physicians support and 17.8% were unsure about supporting CC for SUD and passing a state law (18, 24). The differing physician opinions may be from variance in their patient populations within each specialty or misconceptions about CC SUD laws, with 18.4% of psychiatrists in 2007 unsure whether their state had a CC SUD law for outpatient commitment (18, 24). Responding to rising overdose rates, states with high rates may consider implementing CC SUD laws as a possible solution. Unfortunately, many states that have and use the provision lack a central recording location, making this data inaccessible. One avenue to overcome challenges in measuring CC efficacy for SUD through relapse rates is assessing relapse risk predictors in a group. One study found increased relapse risk predictors, such as reduced social connectedness among sober living residents during the COVID-19 pandemic (26). Another study by Hayaki et al. (27) interviewed 121 individuals prior to CC, then followed them for 3 months after their release. This study showed that 41% used illicit opioids once or more, and over 64% received Medications for Opioid Use Disorder (MOUD) at least once, which was linked to reduced illicit opioid use, demonstrating CC for SUD as an effective prevention modality (28). To further elaborate on the ethics behind CC laws, there is debate that SUD patients may have diminished capacity due to a threat of harm to self or others, or being “gravely disabled,” justifying the use of CC laws. The prefrontal cortex (PFC) regulates the limbic reward system and higher-order functioning, contributing to compulsive drug taking and behavior. Goldstein and Volkow’s (29) study found drug-addicted individuals show PFC dysfunctions associated with increased drug use, poor PFC-related task performance, and greater relapse likelihood. In 2017, Ieong et al. (30) compiled a review describing that 30 chronic heroin users with OUD, after methadone therapy, exhibited reduced functional connectivity between the insula and inferior orbitofrontal cortex, amygdala, putamen, and caudate areas. Individuals addicted to drugs exhibit dorsal anterior cingulate cortex hypoactivity and deficient inhibitory control (30). Chronic self-medication with opioids and methamphetamine results in long-term effects, such as reduced inhibitory control and emotional disruptions persisting beyond short-term abstinence and reemerging with drug-related cues during relapses (30). This supports longer rehabilitation over short abstinence for OUD patients. A 2-week abstinence could improve brain connectivity in the reward system, but impulsivity recovery is lacking (31). Single treatments may not eliminate cravings or provide effective strategies for their transition afterwards, warranting extended care. Moreover, a PFC dysfunction may precede drug usage, increasing SUD vulnerability. The PFC’s role in craving, compulsive usage, and denial needs more study. Furthermore, severe SUD/OUD patients may even display impaired cognitive abilities, impaired logical thinking, and increased impulsivity (13, 14, 25). This shifts the view of CC for SUD to a model of possibly impaired logical thinking and unmanageable lifestyle as SUD or OUD patients exhibit impaired decision-making, although some show increased impulsivity without impaired decision-making (14).

Some limitations to this study include our univariate approach not accounting for confounding factors, such as area poverty level and demographics, and their potential adverse effects on people with SUD. Future research on the association of demographic factors in addition to CC SUD laws is warranted. Previous research found that living in a disadvantaged area, as compared to a prosperous one, was associated with a greater likelihood of both jail sentences longer than 6 months and nonfatal overdoses (13). These confounding factors are worth investigating to compare how area-level deprivation could have similar effects in the U.S. We also did not measure the effectiveness of CC SUD laws on relapse rates which could potentially show the significance of these laws. The data in this study is observational and does not necessarily indicate a causal relationship between the COVID era and OODR. Tracking relapse rates through a dedicated database would offer more accurate data for assessing CC SUD treatment efficacy. However, documenting relapse rates after discharge in this population poses challenges. For instance, the occurrence of relapse and outpatient treatment is often not known or mandated after discharge from a residential treatment facility. Within the past 5 years, some states have enacted CC SUD laws, so there is insufficient time to affect OODR. Moreover, CC laws have been amended to include SUD. Despite these limitations, we found that states with CC SUD laws, after the COVID-era, did not have lower OODR than states without the laws. Future studies should investigate the long-term impact of these laws on opioid overdose and mortality.

## 5 Conclusion

Our study found that overall OODR have increased significantly over the last decade. Contrary to our null hypothesis, we found that the OODR increased at a faster rate post-COVID among the states with CC SUD laws as compared to those without. The CC SUD laws were enacted in response to the opioid epidemic, presumably among the states with high rates of opioid overdose deaths. Future studies on the long-term impact of CC SUD laws on OODR is warranted. We also recommend controlling for the personal level factors such as mental illnesses and area level factors such as poverty and drug possession laws while analyzing the impact of CC SUD laws on OODR.

## Data availability statement

The original contributions presented in the study are included in the article/Supplementary material, further inquiries can be directed to the corresponding author.

## Author contributions

PhC: Conceptualization, Formal analysis, Methodology, Project administration, Validation, Writing – original draft, Writing – review & editing. PeC: Conceptualization, Data curation, Formal analysis, Methodology, Validation, Writing – review & editing. JE: Conceptualization, Investigation, Validation, Writing – original draft, Writing – review & editing. AC: Investigation, Methodology, Resources, Writing – review & editing. HT: Investigation, Validation, Writing – review & editing. RG: Formal analysis, Methodology, Validation, Writing – review & editing. RP: Supervision, Writing – review & editing.

## Glossary

ANOVA: Analysis of Variance
CC: Civil commitment
CDC: Centers for Disease Control and Prevention
dACC: Dorsal anterior cingulate cortex
DEA: Drug Enforcement Agency
ICD-10: International Classification of Diseases, Tenth Revision
IC: Involuntary commitment
MCD: Multiple Cause of Death
MOUD: Medications for Opioid Use Disorder
NFLIS: National Forensic Laboratory Information System
OODR: Opioid overdose death rates
OUD: Opioid use disorder
PDMP: Prescription drug monitoring program
PFC: Prefrontal cortex
rsFC: Resting state functional connectivity
SUD: Substance use disorder
UNODC: United Nations Office on Drugs and Crime
WONDER: Wide-ranging Online Data for Epidemiologic Research
