# Supplementary material for: The impact of civil commitment laws for substance use disorder on opioid overdose deaths
Source: Front Psychiatry. 2024 Feb 2;15:1283169. doi: 10.3389/fpsyt.2024.1283169 (PMC10869443; doi:10.3389/fpsyt.2024.1283169)
Supplement: Supplementary file 1 [file Table_1.docx]

Supplementary Material

The values in Table S1 were used to generate Figure 1. This table shows the annual age-adjusted OODR from 2010 to 2021, comparing states with and without CC SUD laws with confidence intervals.

### TABLE S1. Annual age-adjusted OODR from 2010-2021 comparing states with vs without CC SUD law with confidence intervals.

| ***Year*** | ***No law*** | ***Law*** | ***No law Average CI Lower 95%*** | ***No law Average CI Upper 95%*** | ***Law Average CI Lower 95%*** | ***Law Average CI Upper 95%*** |
| --- | --- | --- | --- | --- | --- | --- |
| 2010 | 14.0281 | 12.5486 | 12.5663 | 15.5219 | 11.3040 | 13.8594 |
| 2011 | 15.5150 | 13.8747 | 13.9825 | 17.0831 | 12.5715 | 15.2179 |
| 2012 | 15.7819 | 13.2563 | 14.2556 | 17.3425 | 11.9740 | 14.5937 |
| 2013 | 16.4363 | 14.1523 | 14.8663 | 18.0431 | 12.8186 | 15.5260 |
| 2014 | 17.9419 | 15.2097 | 16.2819 | 19.6025 | 13.8200 | 16.6514 |
| 2015 | 19.6231 | 16.9869 | 17.9181 | 21.3613 | 15.5143 | 18.4994 |
| 2016 | 22.6950 | 20.6877 | 20.9100 | 24.5144 | 19.0489 | 22.3529 |
| 2017 | 23.6963 | 22.7637 | 21.9300 | 25.4963 | 21.0457 | 24.5026 |
| 2018 | 23.2481 | 21.4906 | 21.5088 | 25.0244 | 19.8223 | 23.1843 |
| 2019 | 23.5544 | 22.9546 | 21.7806 | 25.3644 | 21.2160 | 24.7174 |
| 2020 | 28.2606 | 30.2334 | 26.3331 | 30.2219 | 28.2514 | 32.2269 |
| 2021 | 31.5718 | 35.5854 | 29.5363 | 33.6106 | 33.4351 | 37.7357 |
| Mean | 21.0294 | 19.9787 |  |  |  |  |

### TABLE S2. Source of Variation from two-way ANOVA

| ***Source of Variation*** | ***% of Total Variation*** | ***P value*** | ***P value summary*** | ***Significant?*** |
| --- | --- | --- | --- | --- |
| Interaction | 2.044 | 0.084 | ns | No |
| No Law, Law | 2.535 | 0.054 | ns | No |
| 2010-2019, 2020-2021 | 20.60 | <0.0001 | **** | Yes |

Supplementary Figures S1-S51 display age-adjusted opioid overdose death rates from 2012 to 2021 for all 50 U.S. states and the District of Columbia. The y-axis scale varies in each graph to best capture the annual fluctuations within each state.


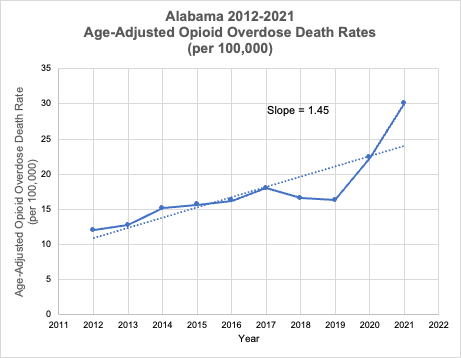


**FIGURE S1.** Alabama 2012-21 OODR


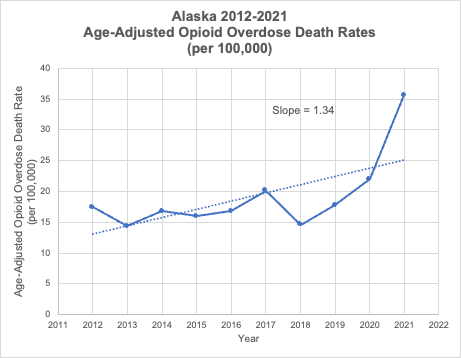


**FIGURE S2.** Alaska 2012-21 OODR


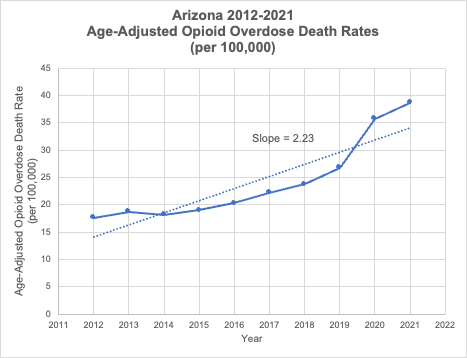


**FIGURE S3.** Arizona 2012-21 OODR


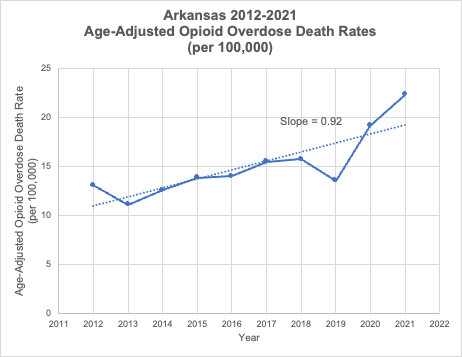


**FIGURE S4.** Arkansas 2012-21 OODR


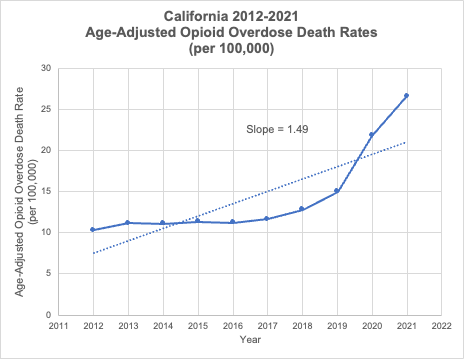


**FIGURE S5.** California 2012-21 OODR


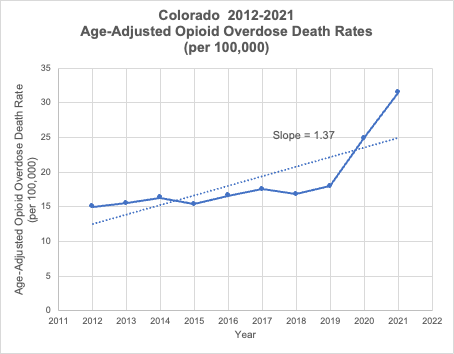


**FIGURE S6.** Colorado 2012-21 OODR


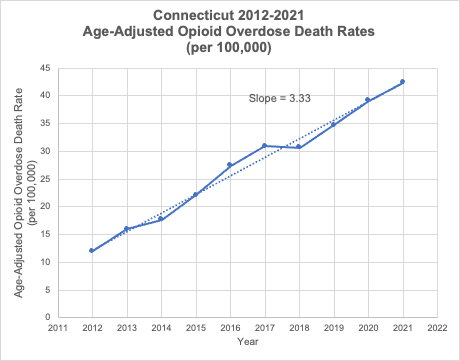


**FIGURE S7.** Connecticut 2012-21 OODR


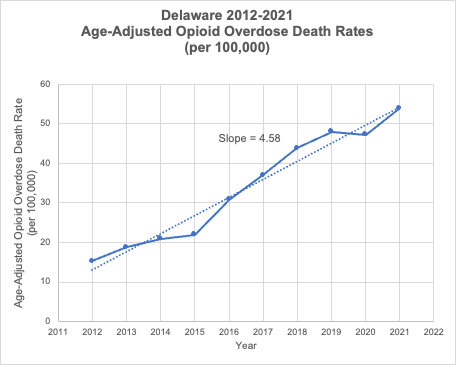


**FIGURE S8.** Delaware 2012-21 OODR


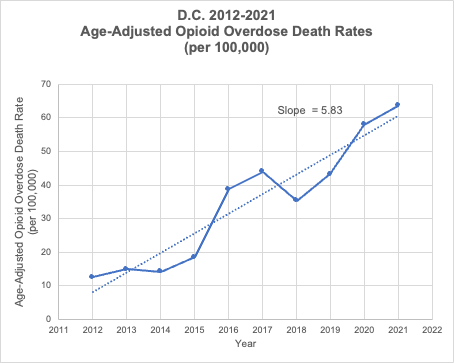


**FIGURE S9.** District of Columbia 2012-21 OODR


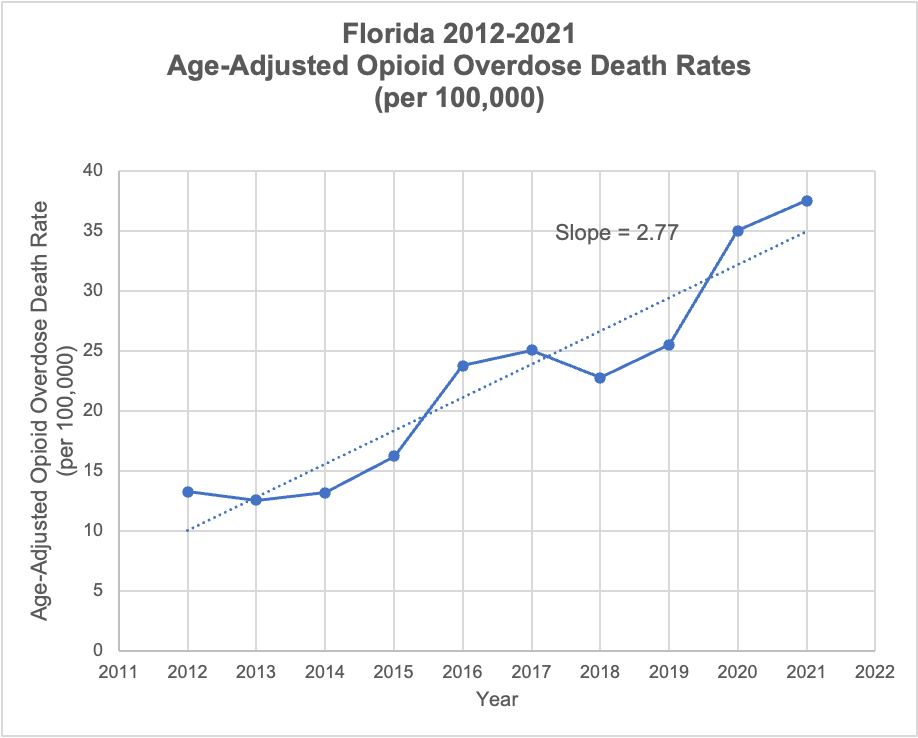


**FIGURE S10.** Florida 2012-21 OODR


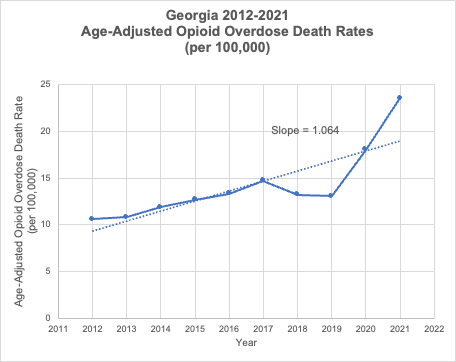


**FIGURE S11.** Georgia 2012-21 OODR


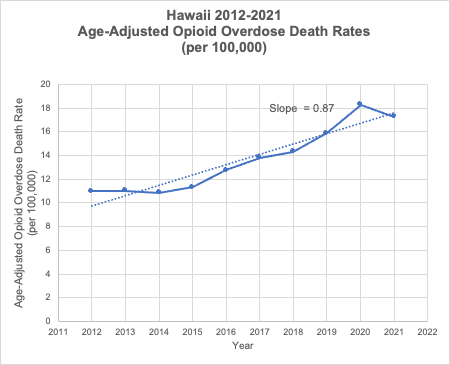


**FIGURE S12.** Hawaii 2012-21 OODR


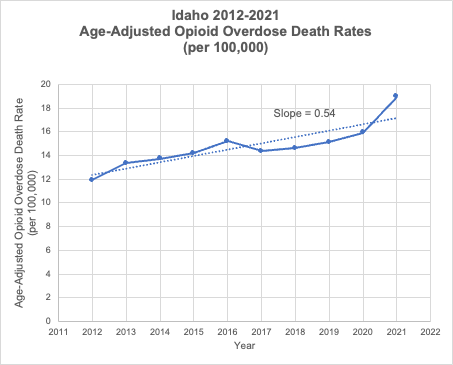


**FIGURE S13.** Idaho 2012-21 OODR


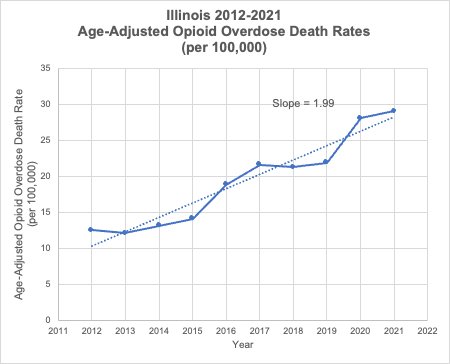


**FIGURE S14.** Illinois 2012-21 OODR


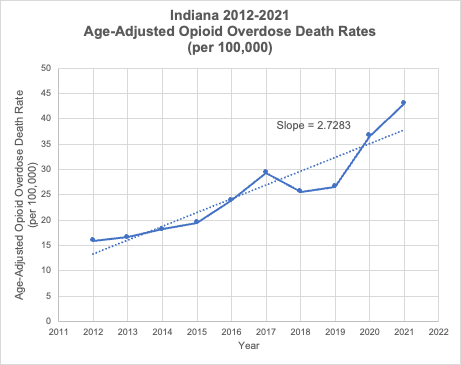


**FIGURE S15.** Indiana 2012-21 OODR


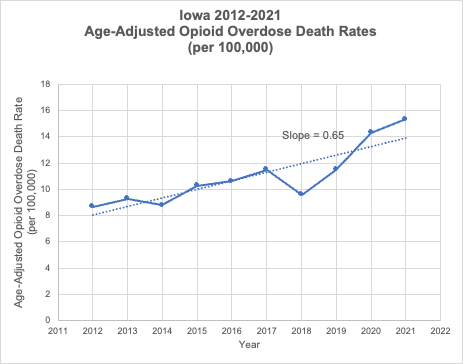


**FIGURE S16.** Iowa 2012-21 OODR


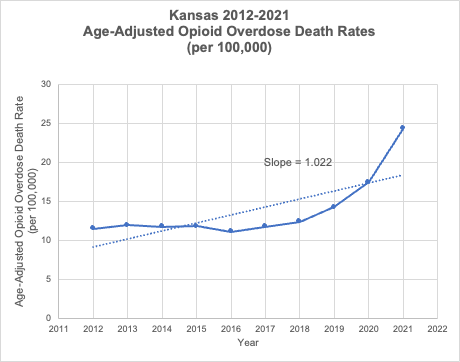


**FIGURE S17.** Kansas 2012-21 OODR


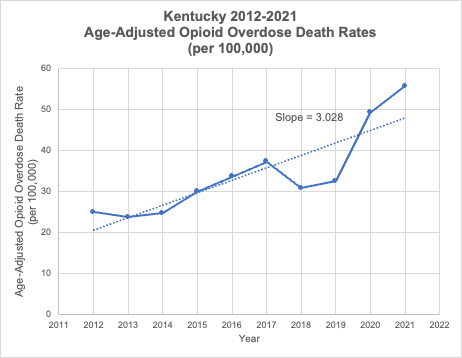


**FIGURE S18.** Kentucky 2012-21 OODR


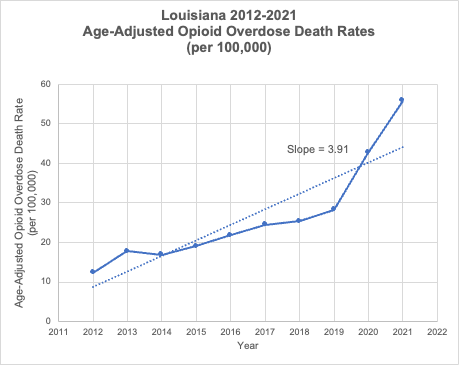


**FIGURE S19.** Louisiana 2012-21 OODR


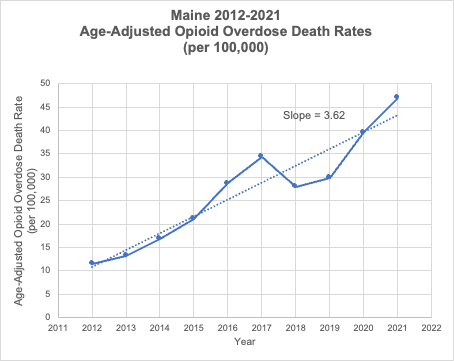


**FIGURE S20.** Maine 2012-21 OODR


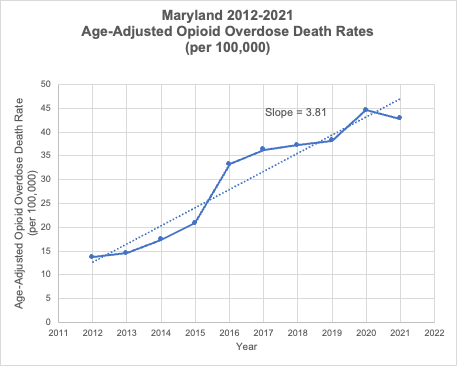


**FIGURE S21.** Maryland 2012-21 OODR


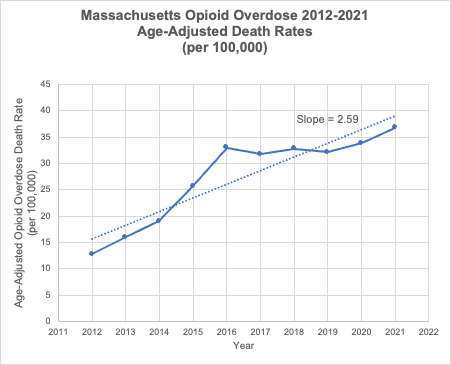


**FIGURE S22.** Massachusetts 2012-21 OODR


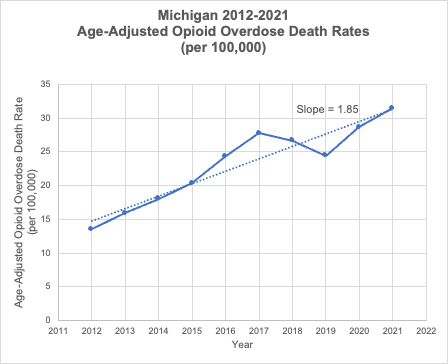


**FIGURE S23.** Michigan 2012-21 OODR


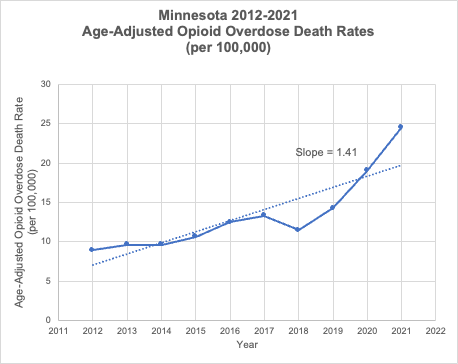


**FIGURE S24.** Minnesota 2012-21 OODR


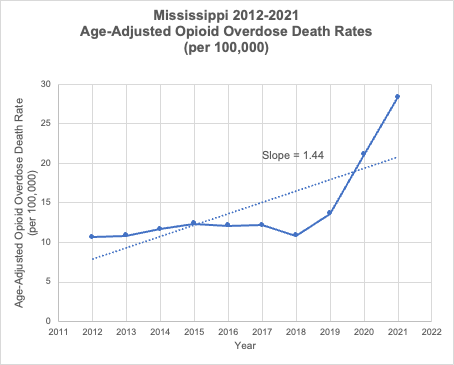


**FIGURE S25.** Mississippi 2012-21 OODR


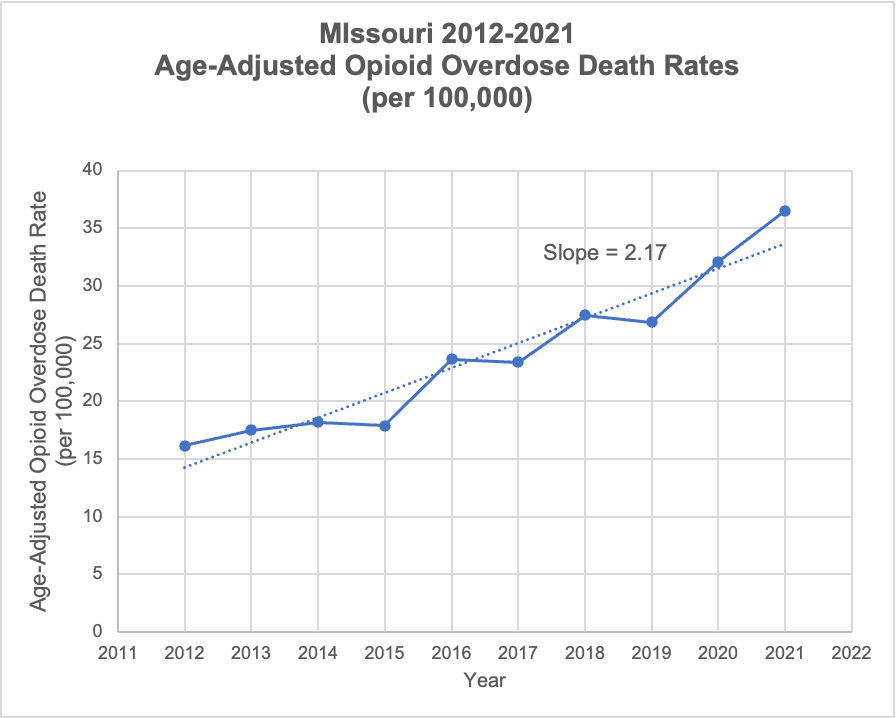


**FIGURE S26.** Missouri 2012-21 OODR


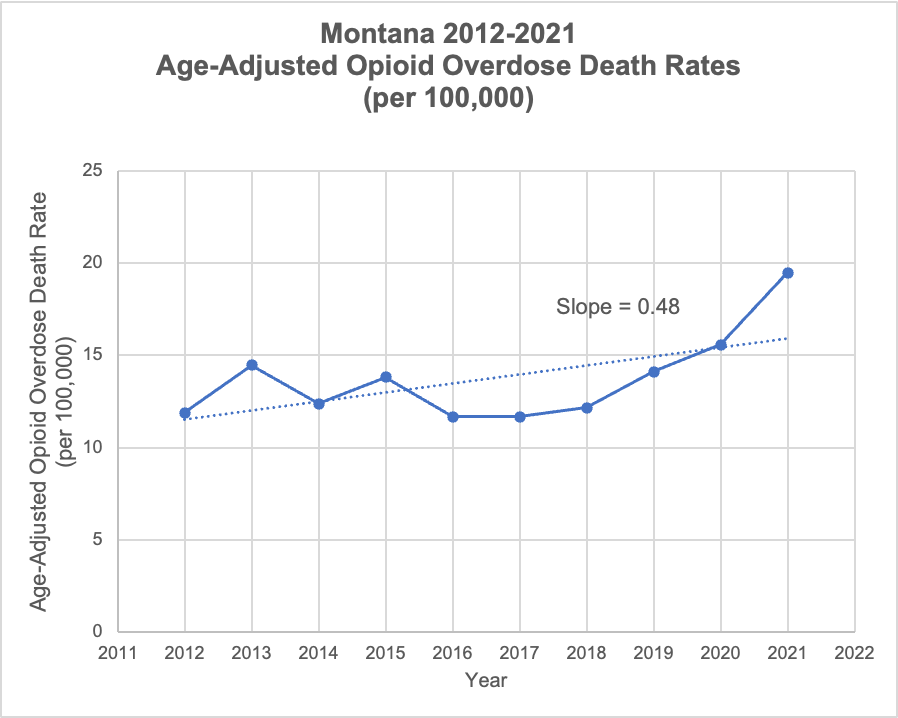


**FIGURE S27.** Montana 2012-21 OODR


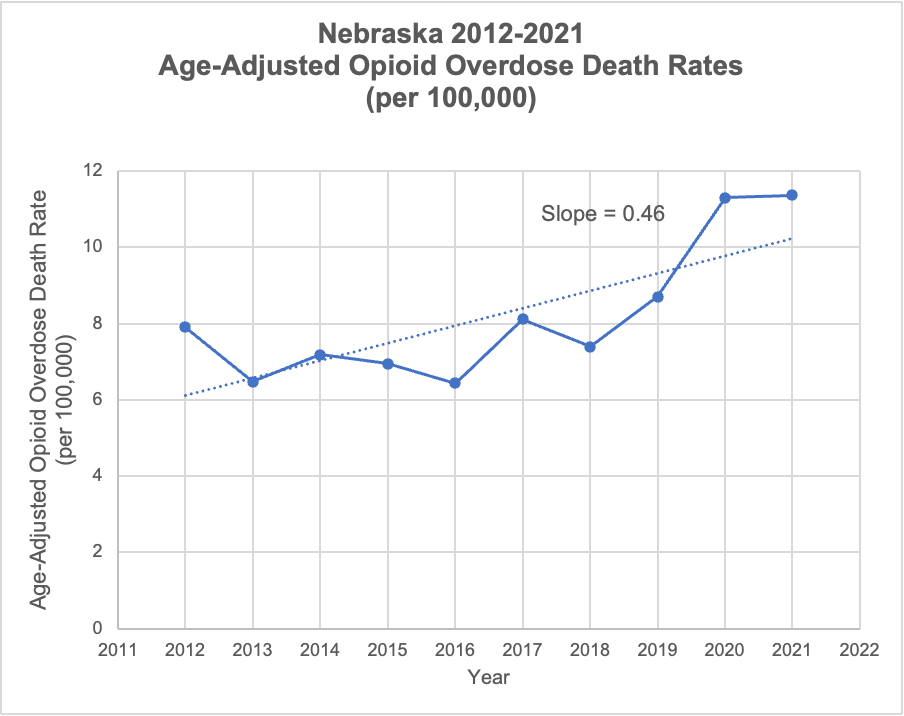


**FIGURE S28.** Nebraska 2012-21 OODR


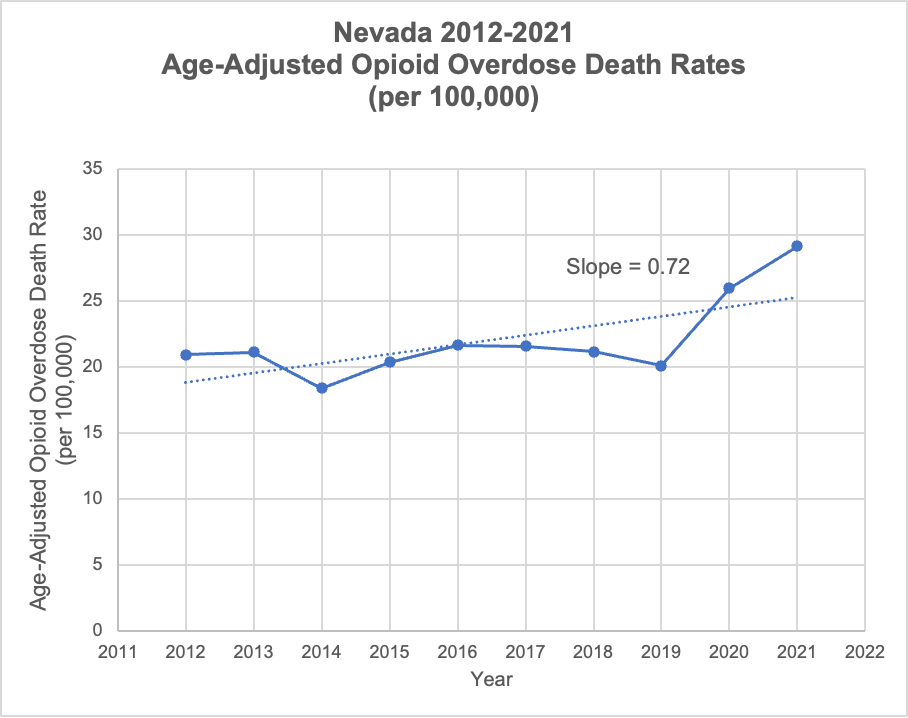


**FIGURE S29.** Nevada 2012-21 OODR


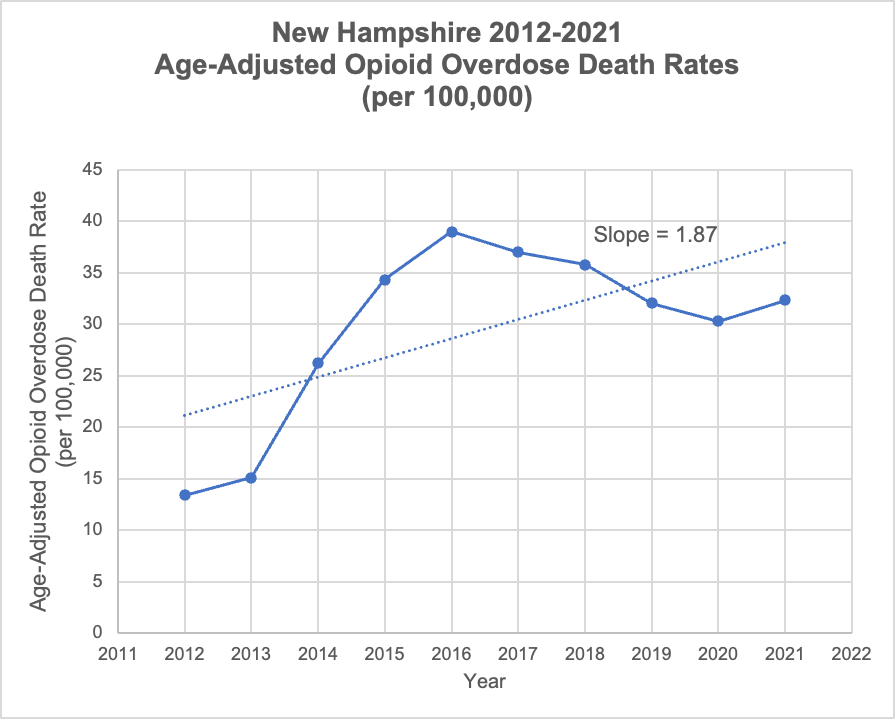


**FIGURE S30.** New Hampshire 2012-21 OODR


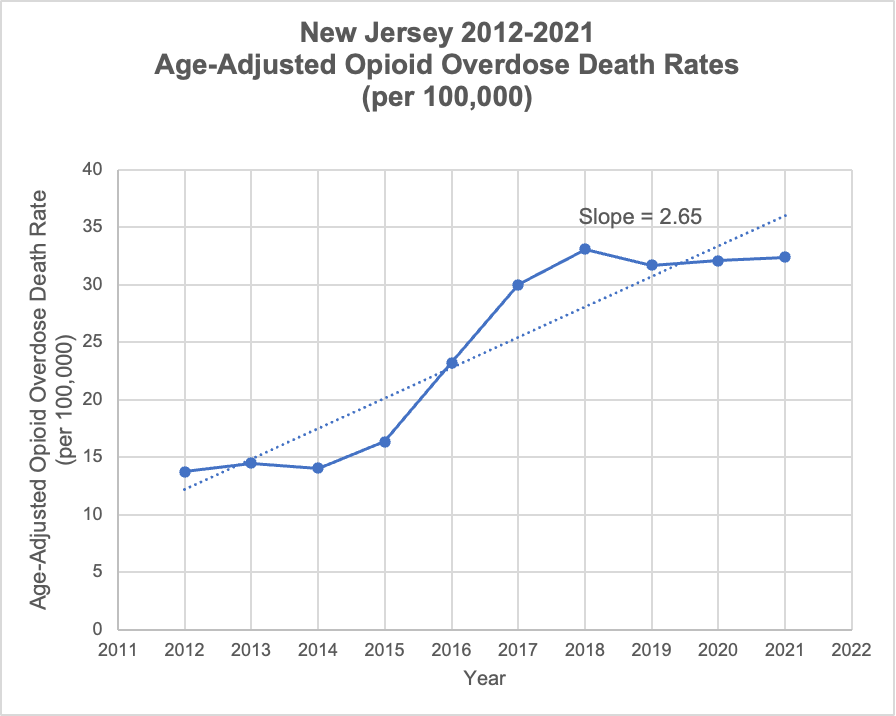


**FIGURE S31.** New Jersey 2012-21 OODR


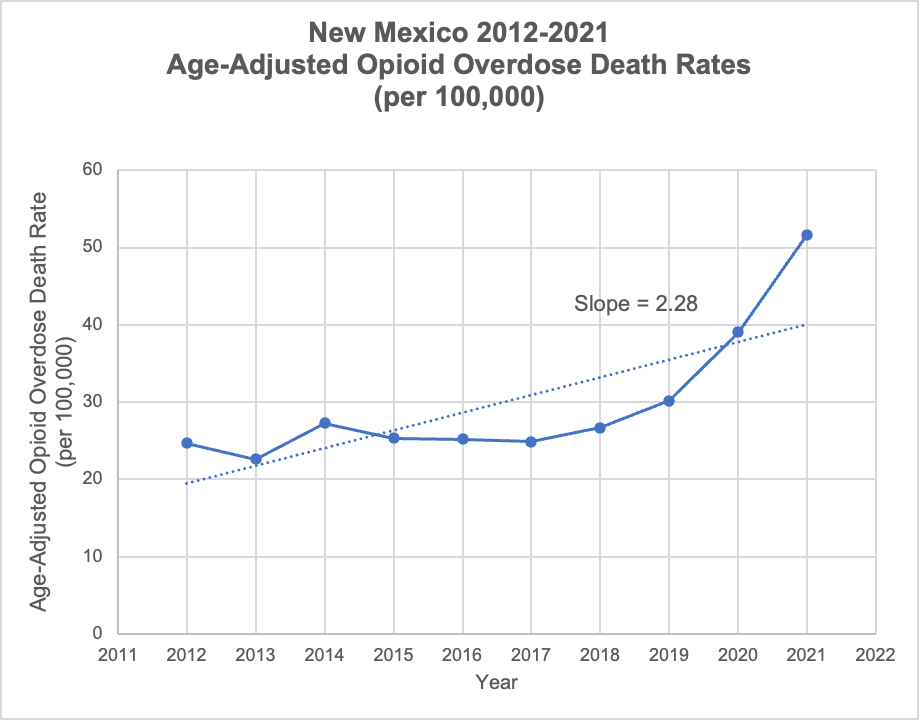


**FIGURE S32.** New Mexico 2012-21 OODR


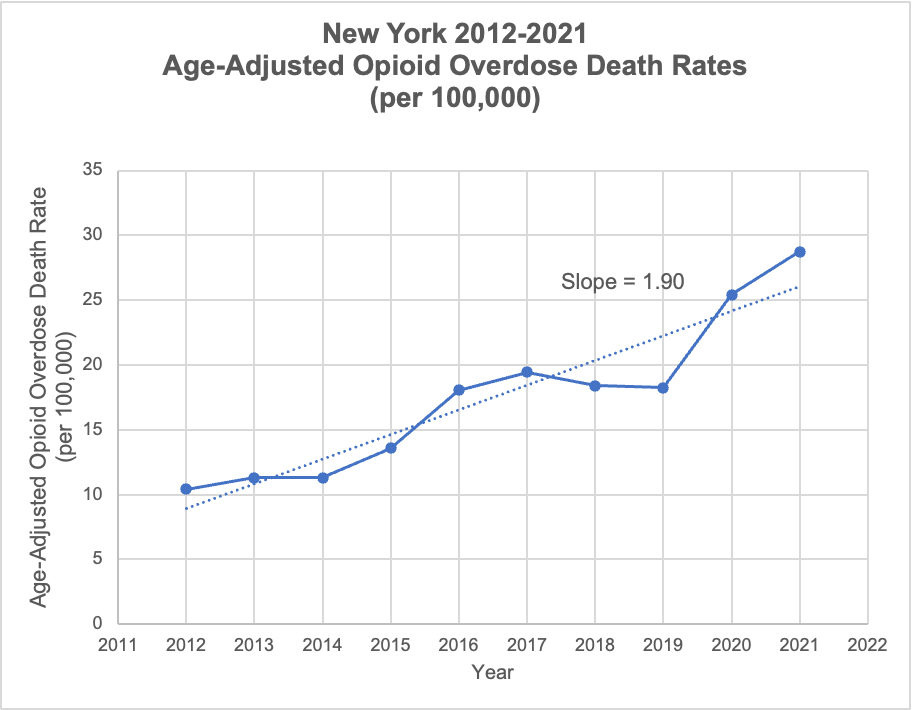


**FIGURE S33.** New York 2012-21 OODR


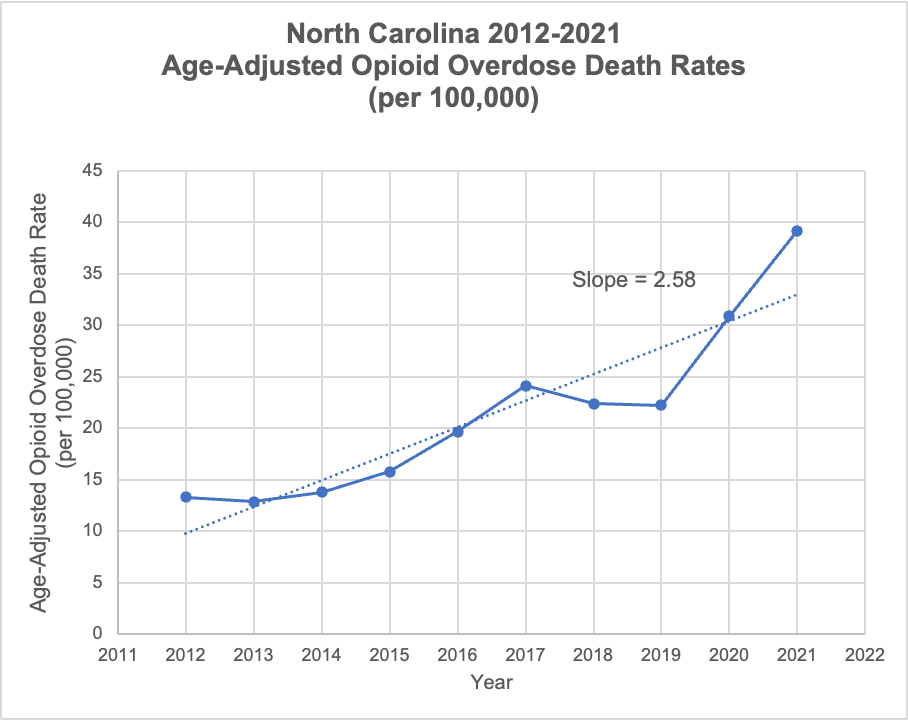


**FIGURE S34.** North Carolina 2012-21 OODR


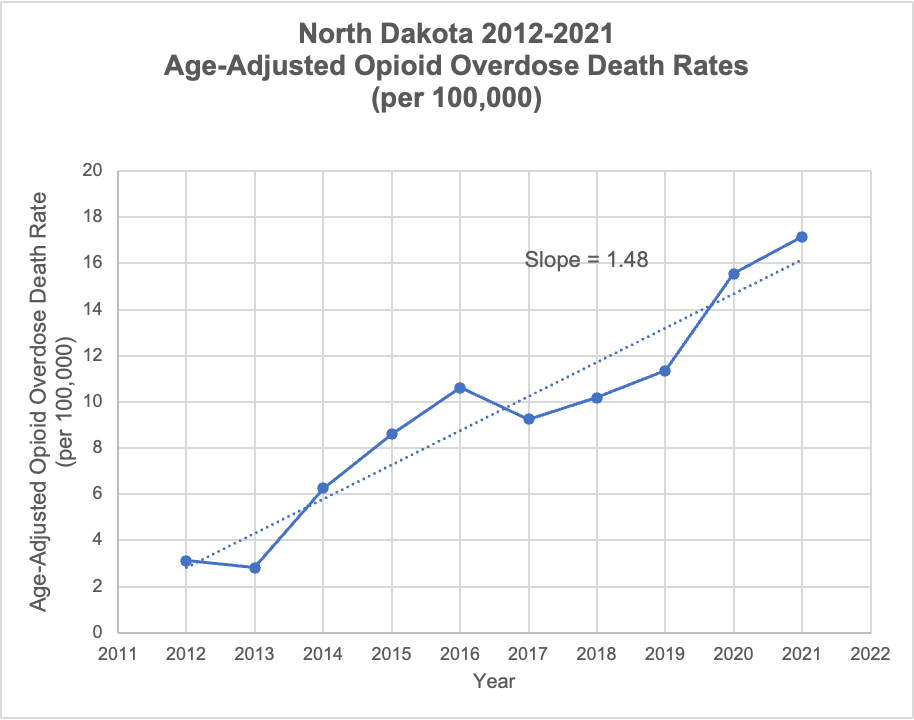


**FIGURE S35.** North Dakota 2012-21 OODR


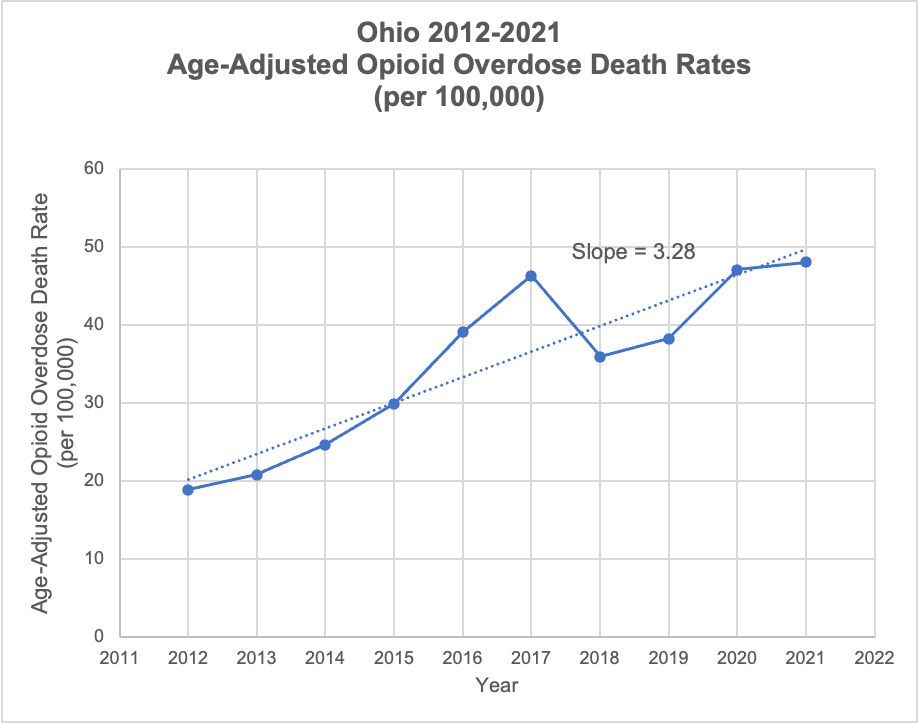


**FIGURE S36.** Ohio 2012-21 OODR


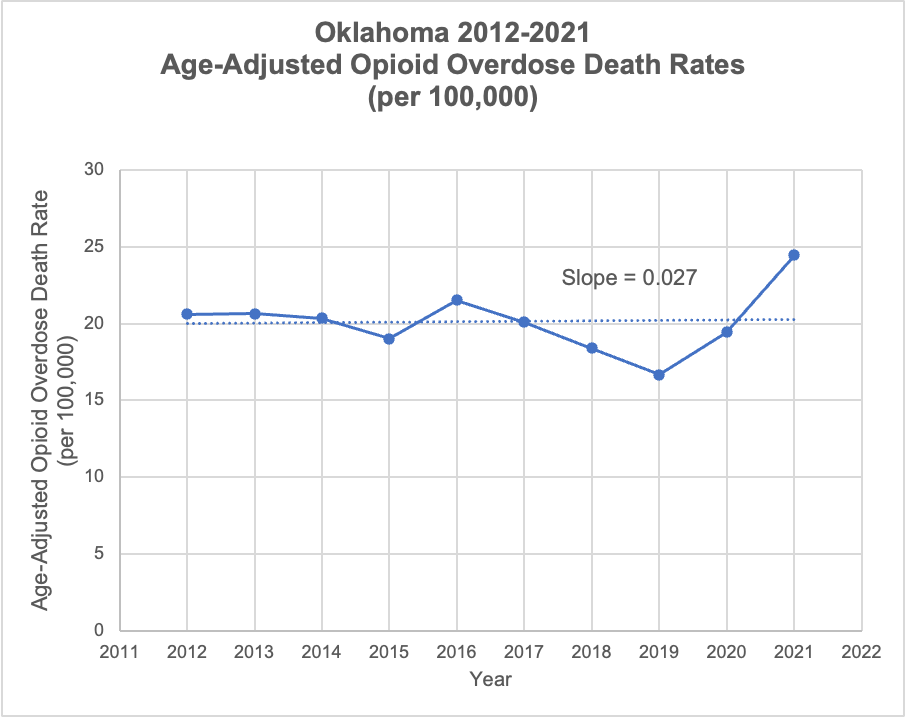


**FIGURE S37.** Oklahoma 2012-21 OODR


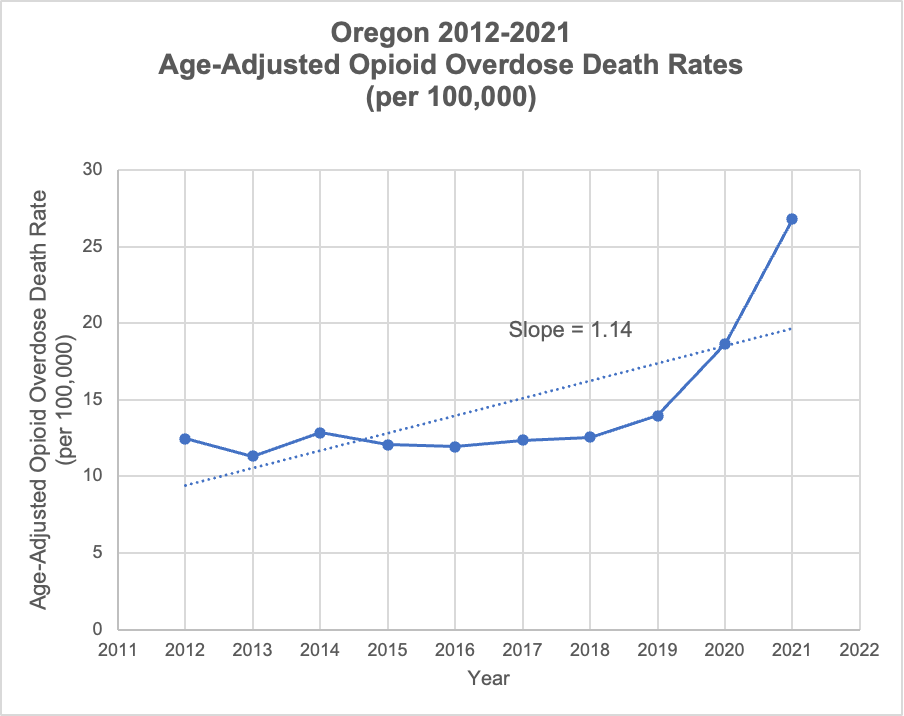


**FIGURE S38.** Oregon 2012-21 OODR


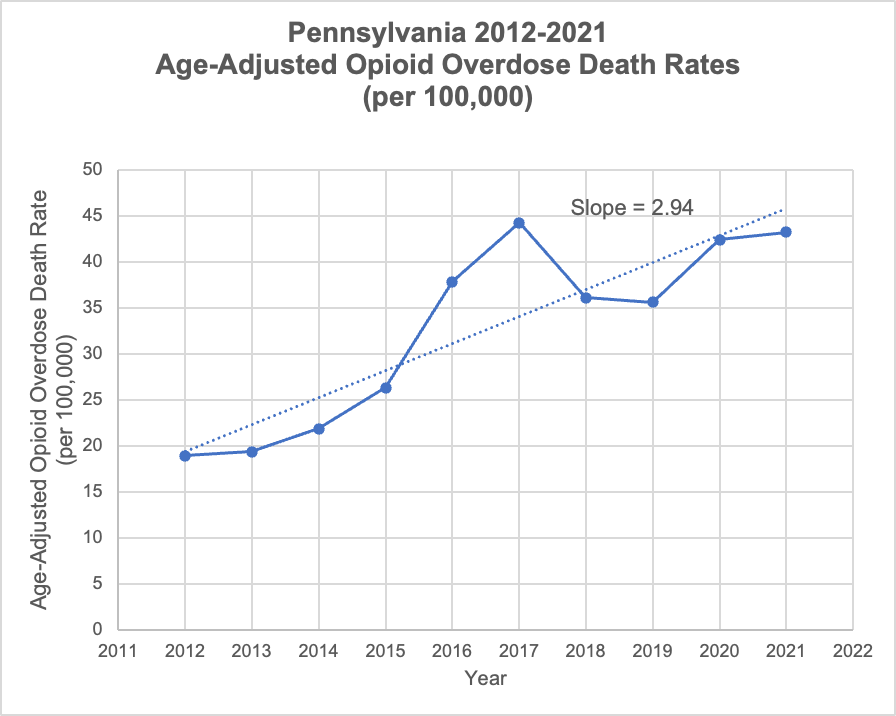


**FIGURE S39.** Pennsylvania 2012-21 OODR


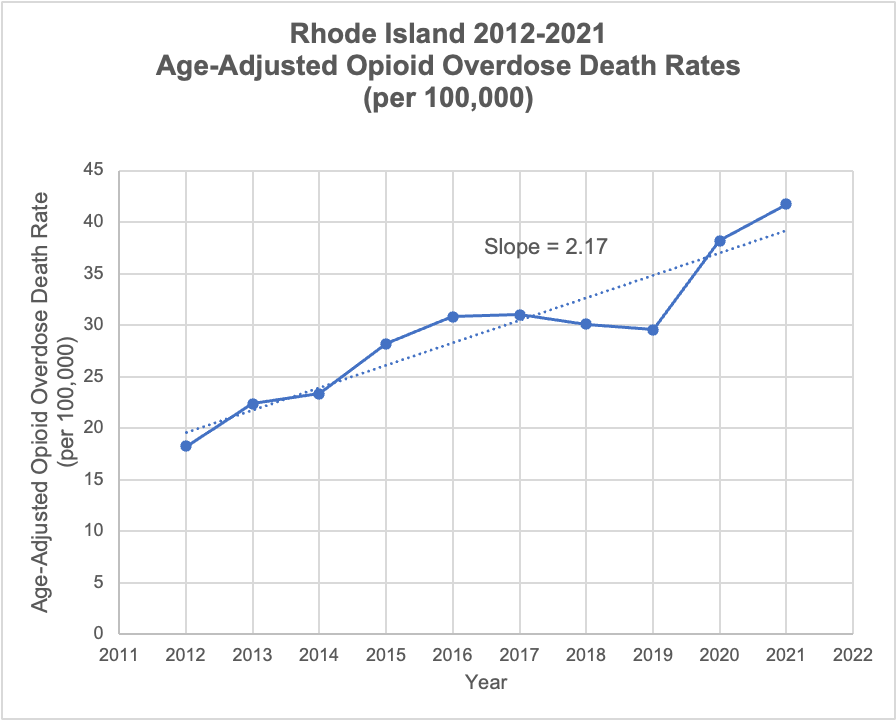


**FIGURE S40.** Rhode Island 2012-21 OODR


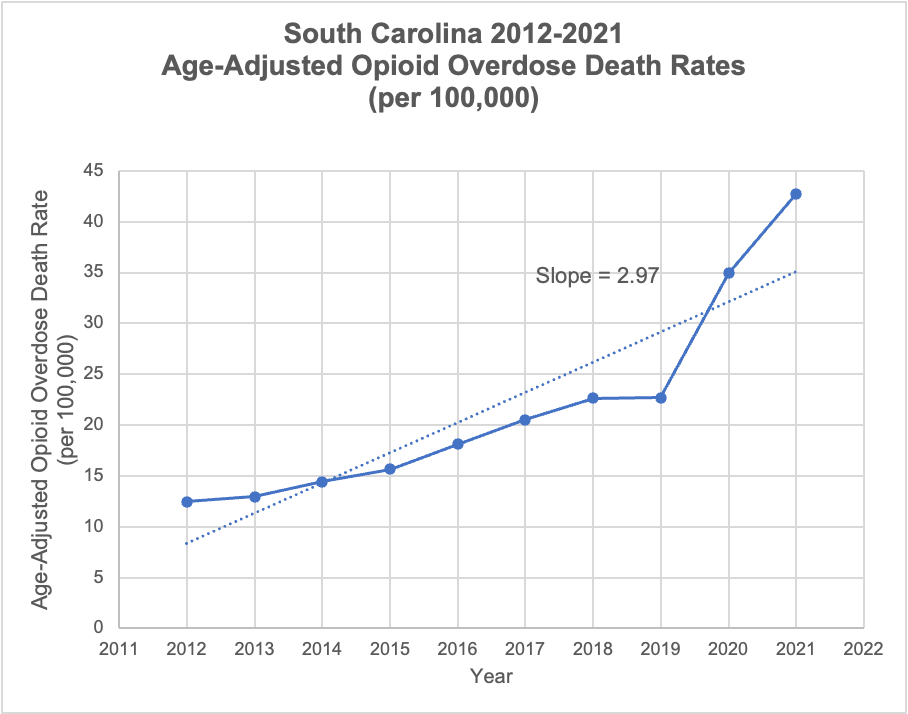


**FIGURE S41.** South Carolina 2012-21 OODR


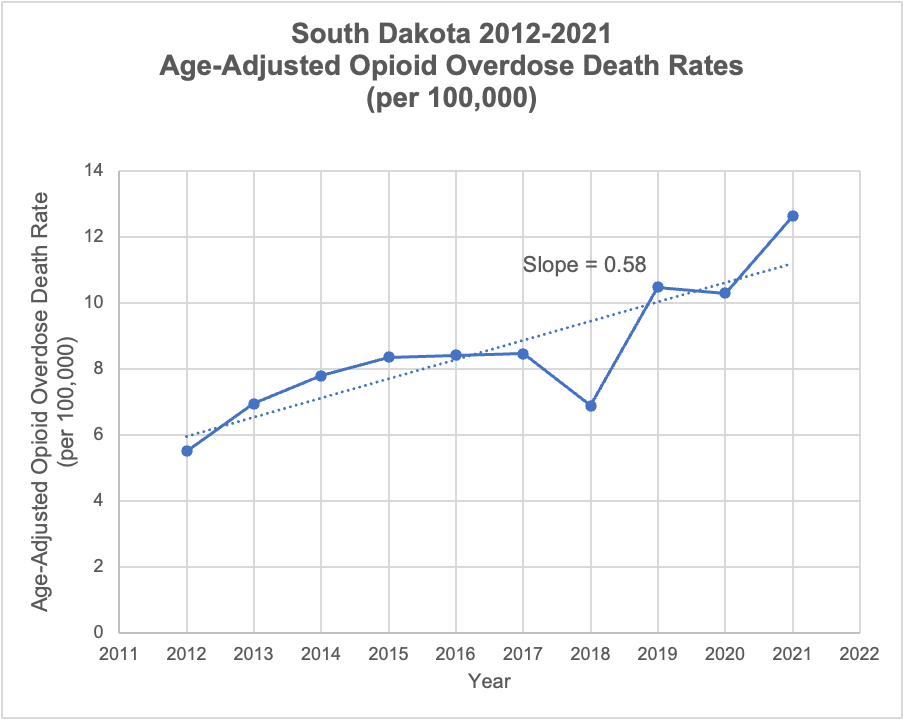


**FIGURE S42.** South Dakota 2012-21 OODR


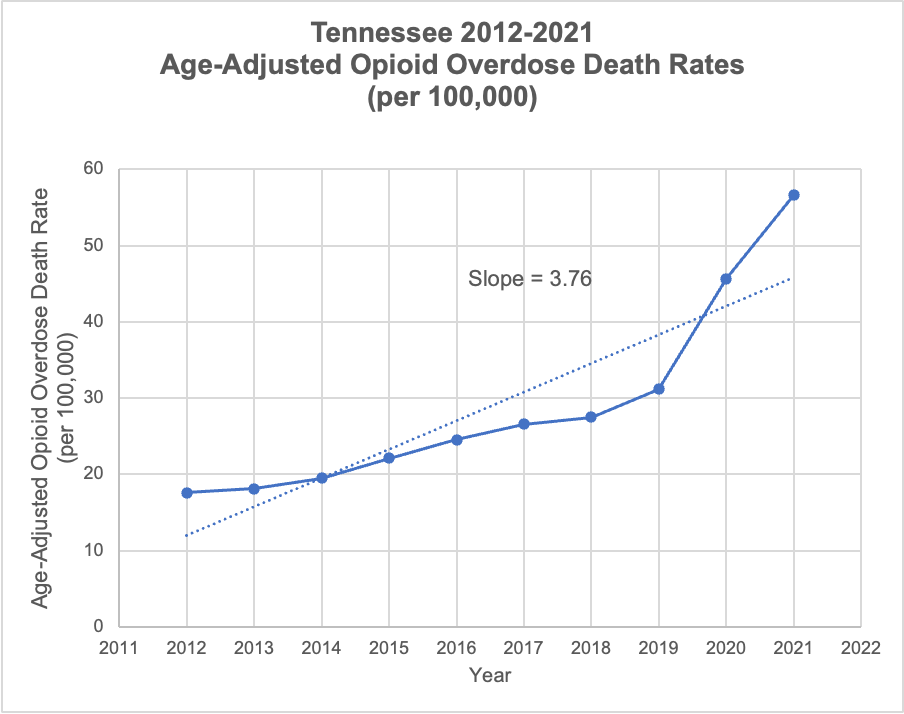


**FIGURE S43.** Tennessee 2012-21 OODR


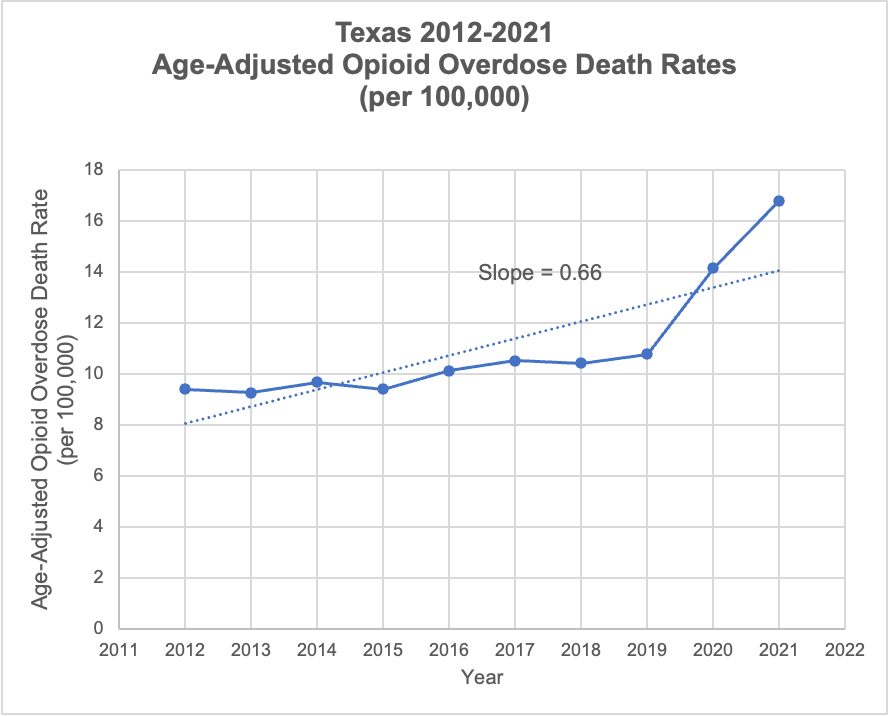


**FIGURE S44.** Texas 2012-21 OODR


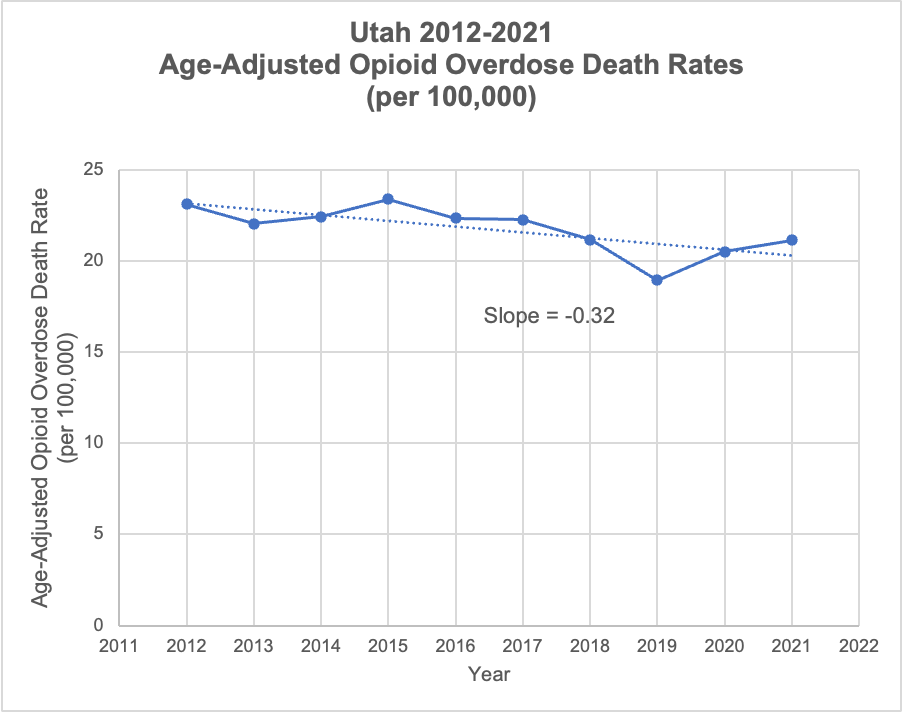


**FIGURE S45.** Utah 2012-21 OODR


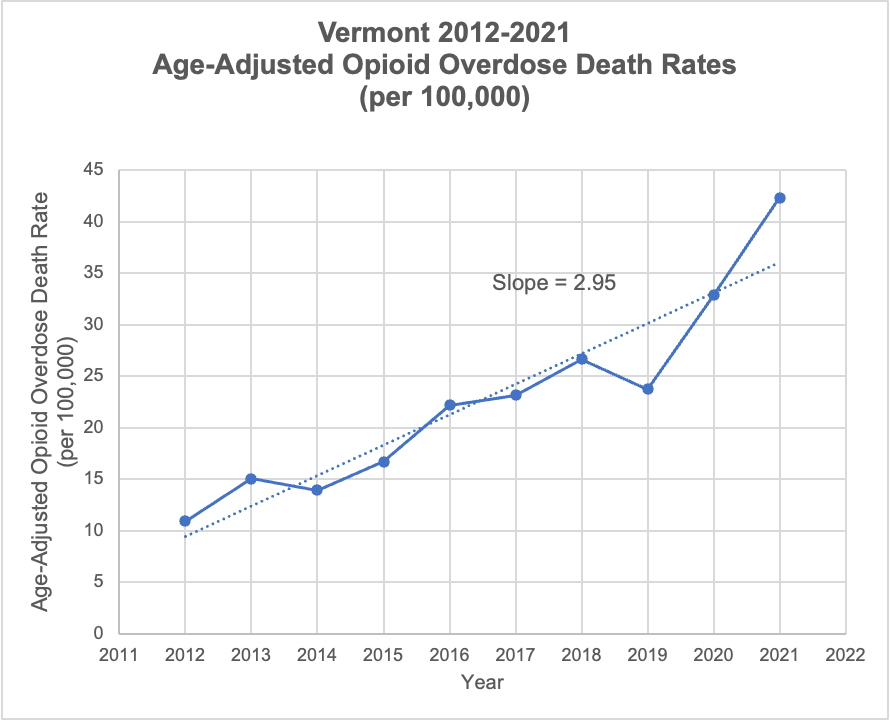


**FIGURE S46.** Vermont 2012-21 OODR


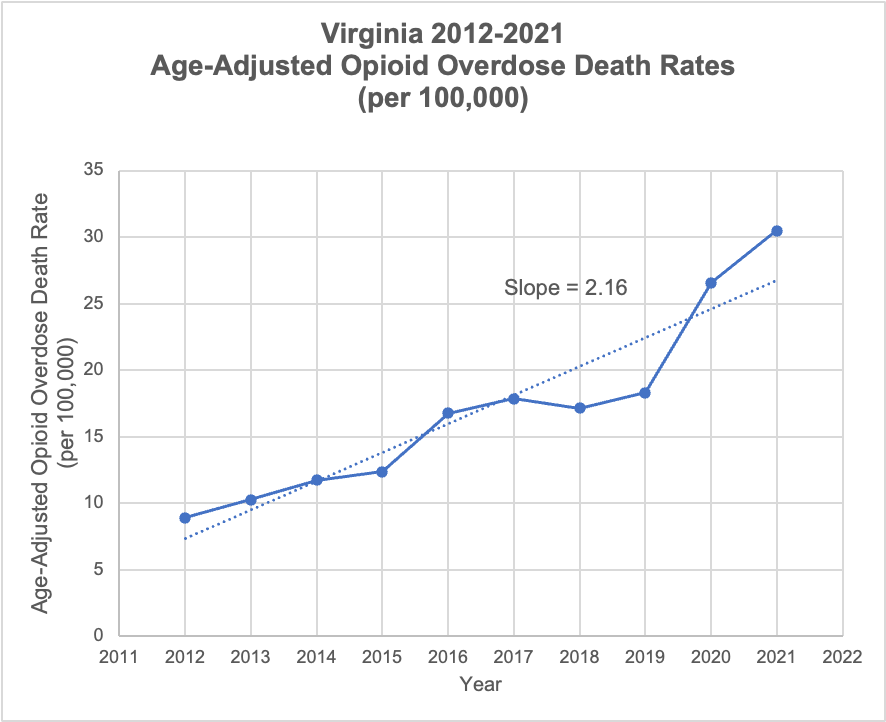


**FIGURE S47.** Virginia 2012-21 OODR


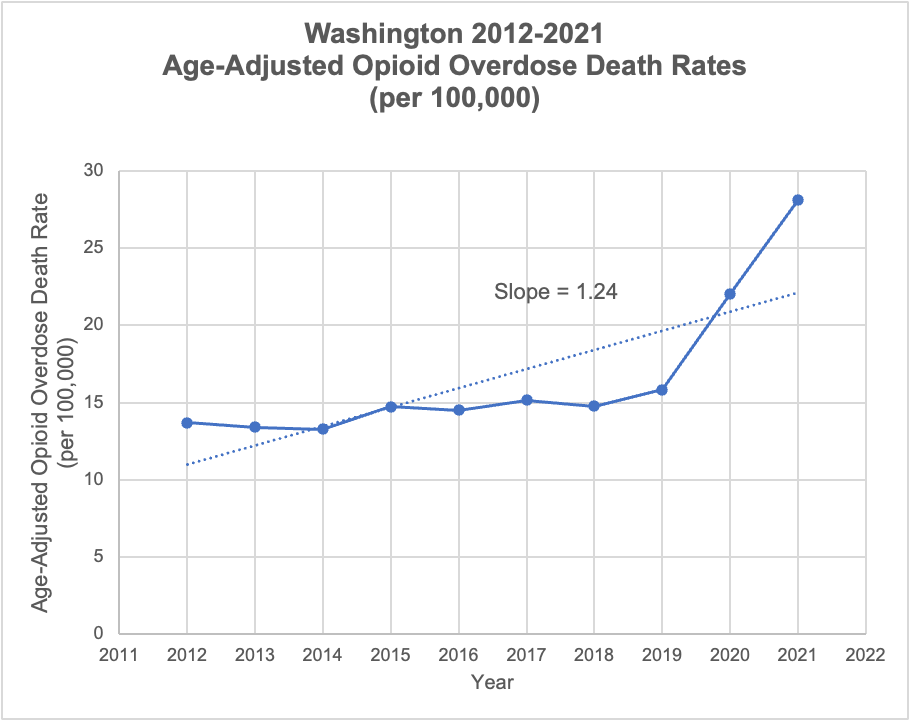


**FIGURE S48.** Washington 2012-21 OODR


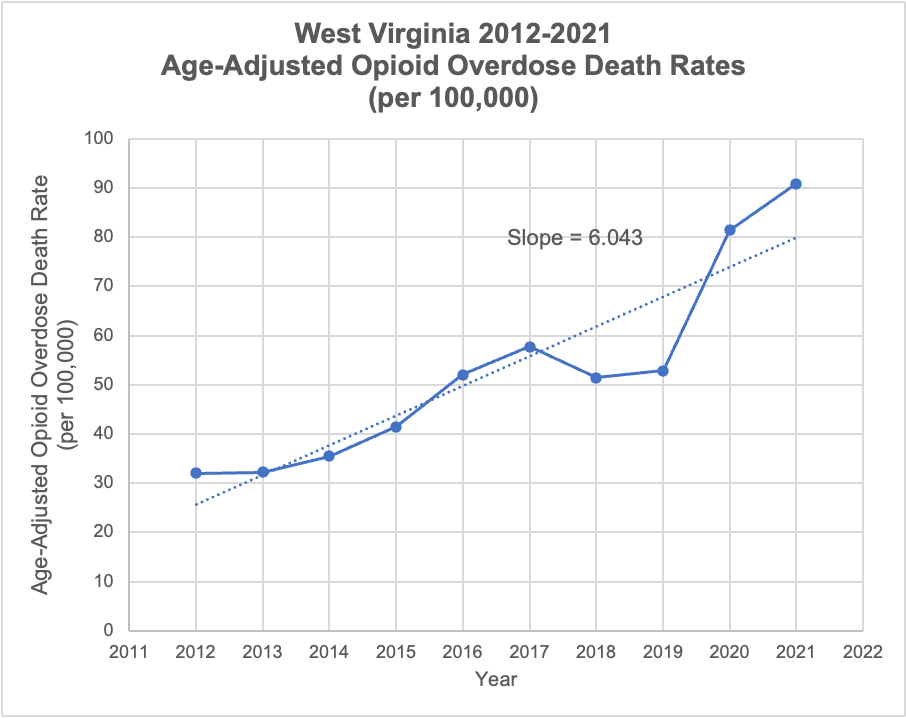


**FIGURE S49.** West Virginia 2012-21 OODR


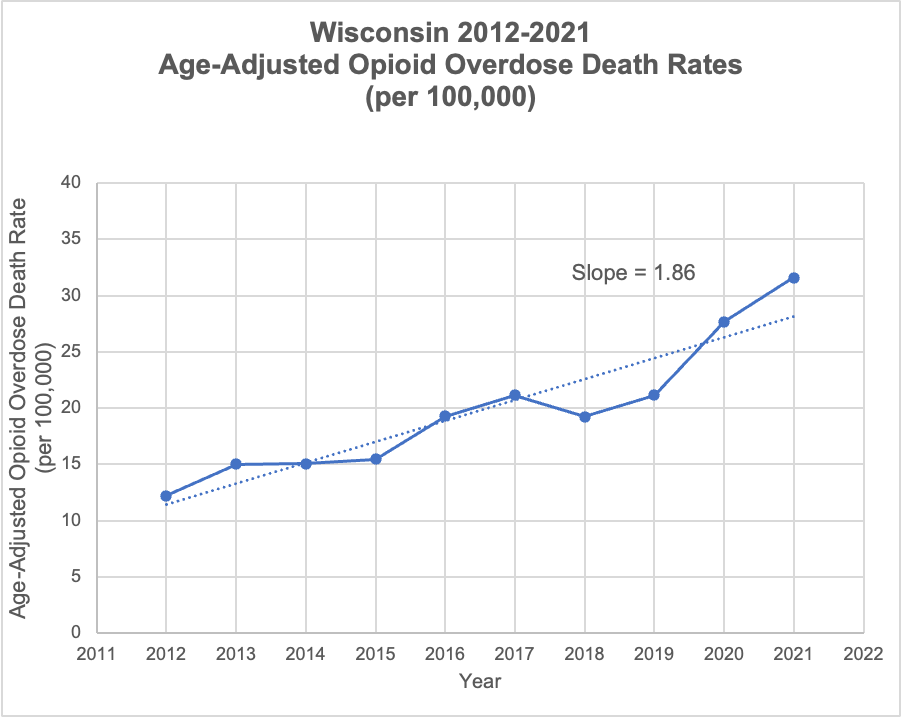


**FIGURE S50.** Wisconsin 2012-21 OODR


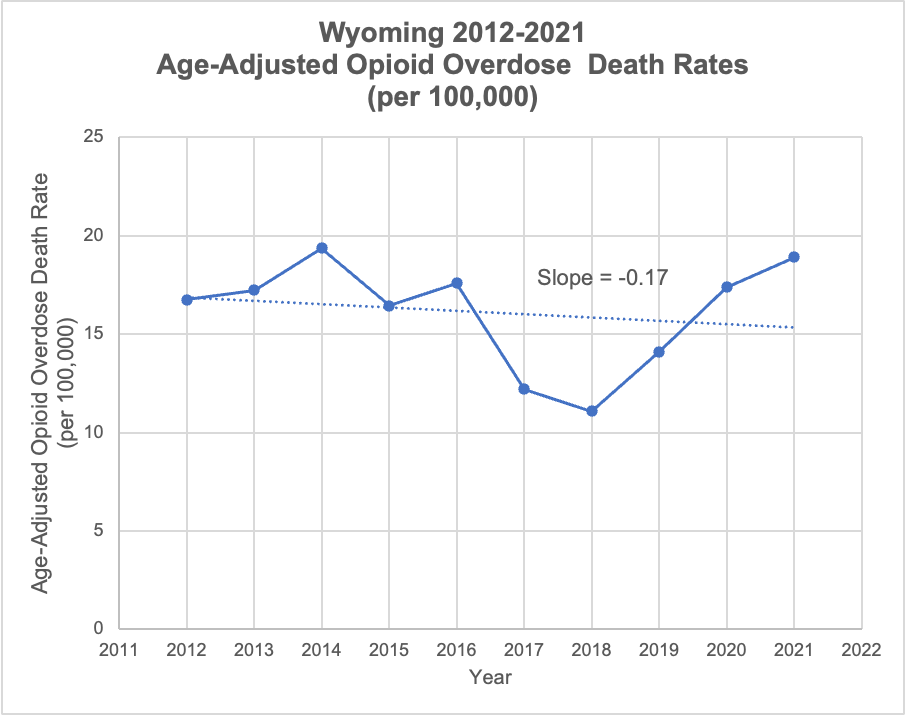


**FIGURE S51.** Wyoming 2012-21 OODR
